# Association of Long-Term Exposure to Traffic-Related Air Pollution with Blood Pressure and Hypertension in an Adult Population–Based Cohort in Spain (the REGICOR Study)

**DOI:** 10.1289/ehp.1306497

**Published:** 2014-02-14

**Authors:** Maria Foraster, Xavier Basagaña, Inmaculada Aguilera, Marcela Rivera, David Agis, Laura Bouso, Alexandre Deltell, Jaume Marrugat, Rafel Ramos, Jordi Sunyer, Joan Vila, Roberto Elosua, Nino Künzli

**Affiliations:** 1 Centre for Research in Environmental Epidemiology (CREAL), Barcelona, Spain; 2 CIBER Epidemiología y Salud Pública (CIBERESP), Barcelona, Spain; 3 Universitat Pompeu Fabra, Departament de Ciències Experimentals i de la Salut (UPF), Barcelona, Spain; 4 University of Montreal Hospital Research Center (CRCHUM), Montréal, Canada; 5 GREFEMA (Grup de Recerca en Enginyeria de Fluids, Energia i Medi Ambient), Girona, Spain; 6 University of Girona (UdG), Girona, Spain; 7 IMIM (Hospital del Mar Medical Research Institute), Barcelona, Spain; 8 Jordi Gol Institute for Primary Care Research (IDIAP-JordiGol), Girona Institute for Biomedical Research (IDIBGI), Catalan Institute of Health, Catalunya, Spain; 9 Department of Medical Sciences, School of Medicine, University of Girona, Girona, Spain; 10 Swiss Tropical and Public Health Institute, Basel, Switzerland; 11 University of Basel, Basel, Switzerland

## Abstract

Background: Long-term exposure to traffic-related air pollution may increase blood pressure (BP) and induce hypertension. However, evidence supporting these associations is limited, and they may be confounded by exposure to traffic noise and biased due to inappropriate control for use of BP-lowering medications.

Objectives: We evaluated the associations of long-term traffic-related air pollution with BP and prevalent hypertension, adjusting for transportation noise and assessing different methodologies to control for BP-lowering medications.

Methods: We measured systolic (SBP) and diastolic BP (DBP) at baseline (years 2003–2005) in 3,700 participants, 35–83 years of age, from a population-based cohort in Spain. We estimated home outdoor annual average concentrations of nitrogen dioxide (NO_2_) with a land-use regression model. We used multivariate linear and logistic regression.

Results: A 10-μg/m^3^ increase in NO_2_ levels was associated with 1.34 mmHg (95% CI: 0.14, 2.55) higher SBP in nonmedicated individuals, after adjusting for transportation noise. Results were similar in the entire population after adjusting for medication, as commonly done, but weaker when other methods were used to account for medication use. For example, when 10 mmHg were added to the measured SBP levels of medicated participants, the association was β = 0.78 (95% CI: –0.43, 2.00). NO_2_ was not associated with hypertension. Associations of NO_2_ with SBP and DBP were stronger in participants with cardiovascular disease, and the association with SBP was stronger in those exposed to high traffic density and traffic noise levels ≥ 55 dB(A).

Conclusions: We observed a positive association between long-term exposure to NO_2_ and SBP, after adjustment for transportation noise, which was sensitive to the methodology used to account for medication.

Citation: Foraster M, Basagaña X, Aguilera I, Rivera M, Agis D, Bouso L, Deltell A, Marrugat J, Ramos R, Sunyer J, Vila J, Elosua R, Künzli N. 2014. Association of long-term exposure to traffic-related air pollution with blood pressure and hypertension in an adult population–based cohort in Spain (the REGICOR study). Environ Health Perspect 122:404–411; http://dx.doi.org/10.1289/ehp.1306497

## Introduction

Air pollution may not only trigger cardiovascular events but promote chronic pathologies and subsequent cardiovascular disease (CVD) (Pope and Dockery 2006), which would further contribute to cardiovascular mortality (Brook et al. 2010). This is supported by animal studies (Brook et al. 2010) and associations between long-term exposure to air pollutants and markers of atherosclerosis (Künzli et al. 2011). Long-term exposure to air pollution may also contribute to CVD through high blood pressure (BP), an established determinant of atherogenesis and CVD, and a leading cause of death (Lopez et al. 2006). Given the ubiquity of air pollution, identifying the association with high BP is important for public health.

There is increasing evidence that short-term exposure to air pollution (i.e., hours to months) is positively associated with systolic BP (SBP) and/or diastolic BP (DBP) levels, although there exists some heterogeneity in previous studies (Brook et al. 2010, 2011; Chuang et al. 2010; Delfino et al. 2010). Less is known about long-term exposure. Four cross-sectional studies in population-based samples, the elderly, or men reported significant positive associations of annual average home outdoor concentrations of different pollutants with BP (Chuang et al. 2011; Dong et al. 2013; Fuks et al. 2011; Schwartz et al. 2012). In contrast, another cross-sectional study in a population-based sample reported inverse associations with SBP (Sørensen et al. 2012). Regarding the prevalence and incidence of hypertension, the few studies available provide inconsistent evidence (Coogan et al. 2012; Dong et al. 2013; Fuks et al. 2011; Johnson and Parker 2009; Sørensen et al. 2012). Plausible biological pathways involve autonomic nervous system imbalance, oxidative stress and systemic inflammation, and subsequent endothelial dysfunction (Brook et al. 2009; Mills et al. 2008).

Several studies suggest that near-road traffic-related air pollution—indicated by residential proximity to traffic—could be particularly important for CVD (e.g., Brook et al. 2009; Hoffmann et al. 2007; Künzli et al. 2010). Traffic-related air pollution could also be relevant for BP (Schwartz et al. 2012). Traffic is also a main source of noise, which has been associated with hypertension in the long-term (van Kempen and Babisch 2012), and may potentially confound or modify the effects of air pollution on BP. However, few studies have adjusted for it (Fuks et al. 2011; Sørensen et al. 2012).

A particular challenge and weakness of previous publications relates to dealing with BP-lowering medication. Conceptually, air pollution would lead to high BP and, subsequently to treatment and lower BP levels. Thus, treatment would be a mediator, not a confounder, of the measured BP levels in those taking BP-lowering medication. Therefore, the common procedure of adjusting for medication may introduce bias, as previously suggested (Fuks et al. 2011; Schwartz et al. 2012; Tobin et al. 2005).

The purpose of this cross-sectional study was to evaluate the association of home outdoor estimates of annual average concentrations of nitrogen dioxide (NO_2_), a marker of traffic-related air pollution, with SBP, DBP, and the prevalence of hypertension, adjusting for traffic-related noise and particularly evaluating different procedures to account for BP-lowering medication. Furthermore, we investigated whether residential proximity to main roads, exposure to high traffic noise levels, and different population characteristics modified the association between pollution and BP. We capitalized on the large and well-defined adult population of the REGICOR (Registre Gironí del Cor) cohort studies conducted in Girona, Spain.

## Methods

*Study population*. The study population consisted of 3,836 individuals, 35–83 years of age, who participated at baseline (years 2003–2006) in one of the population-based cohorts of the REGICOR project, described elsewhere (Grau et al. 2007). Briefly, we randomly selected potential participants from all noninstitutionalized inhabitants of the city of Girona. From those, 73.8% attended the baseline visit. Girona is a typical midsized Mediterranean urban area of nearly 100,000 inhabitants in the northeast of Spain, with a densely populated center where traffic is expected to be the main contributor to air pollution levels. The study was approved by Hospital del Mar Research Institute ethics committee, and participants signed written informed consent.

*Outcomes and health assessment*. Participants fasted for 10 hr before examination. Trained nurses performed the examinations from 0800 to 1100 hours. BP was measured at the beginning, following the Joint National Committee VII recommendations (Chobanian et al. 2003), in sitting position, and with a calibrated automatic device (OMRON 711; Omron Healthcare, Lake Forest, IL, USA) that also registered heart rate. The first and second measurements were done after at least 10 and 3 min of rest, respectively. If measurements differed by ≥ 5 mmHg, a third measurement was taken. To avoid “white coat effect,” we used the last measurement available. The nurses also withdrew blood to obtain cholesterol, triglyceride, and fasting glucose levels.

We collected individual questionnaire information on smoking, weekly leisure time physical activity (based on the Minnesota Leisure-time Physical Activity Questionnaire) (Elosua et al. 2000), daily alcohol intake, living alone, family history of cardiovascular deaths, diet (defined with the REGICOR score for adherence to Mediterranean diet) (Schröder et al. 2004), educational level, and occupational status. In addition, we assessed socioeconomical status (SES) with the deprivation index at the census tract level (Domínguez-Berjón and Borrell 2005). We defined diabetes as fasting blood glucose level ≥ 126 mg/dL or reported treatment with antidiabetic drugs, body mass index (BMI) as weight/height squared (kilograms per meter squared), and cardiovascular disease as having ever had a cardiovascular event (myocardial infarction or stroke) or cardiovascular-related surgery intervention.

We defined hypertension as having SBP or DBP ≥ 140 or 90 mmHg (Chobanian et al. 2003), respectively, or as taking antihypertensive treatment, reported with a positive response to the question “Do you take or have you taken any doctor-prescribed medication to reduce blood pressure in the last two weeks?” For BP analysis, we accounted for any “BP-lowering medication.” It included antihypertensive treatment, as defined with the question above, or the use of any treatment from the medication list provided by participants and coded by a physician as “antihypertensive” or “beta-blocker” [i.e., diuretics, ACE (angiotensin-converting-enzyme) inhibitors, alpha- or beta-blockers, angiotensin receptor II blockers, and calcium channel blockers].

We used two alternative definitions of hypertension in sensitivity analyses to evaluate the potential misclassification of cases: *a*) We considered the antihypertensive-like treatment of the medication list in the hypertension definition instead of the self-reported antihypertensive treatment described above, and *b*) we excluded participants with BP levels close to the cut-off value for hypertension (i.e., we excluded nonmedicated participants with SBP and DBP ≥ 135/85 mmHg and < 150/95 mmHg). Besides, we defined “hypertension or prehypertension,” which included both hypertensives and prehypertensives, as cases. Prehypertensives are commonly classified as nonhypertensive and are not medicated; however, they have nonoptimal BP levels (i.e., SBP or DBP ≥ 120/80 mmHg but below the cut-off for hypertension) (Chobanian et al. 2003) that could be influenced by air pollution.

*Exposure assessment*. We geocoded participants’ residential addresses at enrollment. We estimated annual average outdoor NO_2_ (micrograms per cubic meter) at the residences with a city-specific land use regression (LUR) model (*R*^2^ = 0.63) based on a dense network of residential outdoor NO_2_ measurements (years 2007–2009), as described elsewhere (Rivera et al. 2013). The main predictor variables were the height of the sampler and traffic-related variables at different radius buffers (from 25 to 1,000 m) around the sampling locations.

To control for acute effects of short-term air pollution and temperature on measured BP, we obtained daily means of temperature and of NO_2_ concentrations at an urban background station from the regional air quality monitoring network. Season was categorized as winter (January–March), spring (April–June), summer (July–September), and autumn (October–December).

We also derived long-term average traffic noise levels [A-weighted decibels; dB(A)] 2 m from the façades and at the floors’ height of each dwelling with a detailed and validated city-specific noise model (year 2005). This model complies with the European Noise Directive 2002/49/EC (END) and uses the interim European method NMPB routes-96 [CERTU (Centre d’Études sur les Réseaux, les Transports, l’Urbanisme et les Constructions Publiques) et al. 1997]. Estimates were computed at each receptor point by numerical calculations using CadnaA software (DataKustik, Greifenberg, Germany). The main input variables were street slopes, type of asphalt, urban topography, and traffic density. Because railway noise has also been associated with BP (Dratva et al. 2012), and a single North–South rail bridge crosses dense traffic areas in the city, we also derived residential railway noise estimates from an END-based model according to the International Organization for Standardization (ISO; Geneva, Switzerland) standard 9613. The propagation model was built upon source identification of railway noise. This consisted of daytime and nighttime noise measurements of frequencies (in one-third octave bands) and equivalent levels [in dB(A)] of freight and normal trains (a total of 72 measurements). Measurements were taken with a SC-30 sound level meter and a CB-5 calibrator (CESVA, Barcelona, Spain). Because it has been suggested that transportation noise may affect cardiovascular health particularly during the restorative sleep processes at nighttime (Jarup et al. 2008), we used the nighttime (2300–0700 hours) noise indicators (*L*_night_). The small airport located outside the city did not affect our study population.

Traffic markers (traffic intensity at the nearest road and traffic load within a 500-m buffer) were collected using the city road network with linked traffic intensities from local registries and traffic (Rivera et al. 2012).

*Statistical analyses*. We excluded participants with missing information (*n* = 101) on the outcomes, exposure, or main model covariates. We assessed bivariate associations with Spearman rank correlation between continuous variables, with the chi-square test between categorical variables, and with the Kruskal–Wallis test between continuous and categorical variables. We used multivariate linear regression models for BP and logistic regression for hypertension, and performed regression diagnostics. The shape of the association between NO_2_ and the outcomes was depicted using smooth splines with multivariate generalized additive models. Inclusion of covariates was based on the hypothesized causal pathway of long-term effects of NO_2_ on BP and current evidence (Fuks et al. 2011). We also built saturated models with all covariates univariately associated with the outcome and exposure (*p* < 0.2) and performed backward regression, manually removing the variables with the highest *p*-value one-by-one until estimates changed ≥ 20%. If changes were smaller, we stopped when all *p*-values were < 0.1. Finally, we assessed the inclusion of potential intermediate variables, co-morbidities, and use of different lags for daily temperature and NO_2_ levels before examination (lags: 1, 2, 3, and the average from 0 to 3 days), compared with lag 0 (used by default). Temporal trend (day of examination) was also examined to control for potential decreasing trends in BP levels over the study period due to improved BP management. Because the different model specifications gave similar results (data not shown), we present final estimates with the most parsimonious model based on background regression (i.e., a model with the minimum set of covariates providing the same point estimates as did more complex models), to avoid overadjustment and variance inflation. The models were adjusted for the following variables: age, age squared, sex, living alone, educational level, diabetes, BMI, nighttime railway noise, road traffic noise, smoking, alcohol consumption, deprivation, daily NO_2_ (lag 0), and temperature (lag 0). We categorized BMI and introduced a square term for age to meet linearity with the outcomes. Nonlinearity was assessed *a priori* with univariate generalized additive models, checking splines, and was later tested in regression diagnostics.

A main objective of this study was to rigorously investigate potential biases related to different methodologies used or proposed to control for BP-lowering medication. Thus, we estimated the effects of NO_2_ on BP as follows: *a*) restricting analysis to participants not taking any BP-lowering medication (i.e., in “nonmedicated”); *b*) restricting analysis to “medicated” participants; *c*) ignoring treatment; *d*) adjusting for treatment; *e*) adding a fixed value of mmHg for SBP (+10, 15, and 20) and DBP (+5, 10, 15) to those treated; and *f*) using censored normal regression. This method models the untreated BP levels assuming they are normally distributed. In the medicated participants, the untreated BP levels are not observed, but they are assumed to be at least as high as the measured levels (right censoring). The model effectively assumes that, given the covariates, untreated BP levels above a certain value have the same distribution in medicated and non-medicated participants. Methodologies *a*–*d* were used for comparison with previous literature. Methodologies *e* and *f* were favored in a comprehensive simulation study, but rely on nonmeasurable assumptions that might be violated in our study population (Tobin et al. 2005).

We assessed effect modification of the association between NO_2_ and BP among nonmedicated participants by levels of traffic noise at night [*L*_night_ < 55 dB(A) vs. ≥ 55 dB(A)], traffic intensity at the nearest road, and traffic load within 500 m as binary variables categorized at the median. We also evaluated age, sex, educational level, smoking, Mediterranean diet, living alone (a marker of uncontrolled hypertension) (Morgado et al. 2010), diabetes, CVD, and season of examination (given possibly larger effects in summer (Fuks et al. 2011; Sørensen et al. 2012). Effect modification was tested by adjusting for an interaction term (i.e., NO_2_ × evaluated categorical variable) and checking its statistical significance (i.e., *p* for interaction) as well as the studied association per categories of the tested variable. The nonmedicated group was of primary interest because the outcome is not influenced by BP-lowering medication in these participants. For comparison, we further evaluated these interactions in the entire sample, using methods *e* (adding 10–15 mmHg for SBP and 5–10 mmHg for DBP to measured values) and *f* (censored regression) to control for medication. Finally, we tested whether associations for nonmedicated and medicated were statistically different by introducing an interaction term (BP-lowering medication × NO_2_) in the model using method *d*) (adjusting for treatment).

We also performed sensitivity analyses for residential mobility by restricting analyses to subjects not moving residence in the preceding 2, 5, and 10 years, and checked the time window of exposure by using the average NO_2_ exposure of the preceding 10 years for a subset of 2,402 individuals with information on residential history.

Estimated effects on BP and hypertension are expressed per 10-μg/m^3^ increase in NO_2_ unless differently specified. We defined statistical significance at an alpha level of 0.05.

Analyses were done using Stata version 12.0 (StataCorp, College Station, TX, USA) and R version 2.12 (http://www.r-project.org/).

## Results

The final sample size consisted of 3,700 individuals. Participants excluded from the study had slightly higher SBP levels, less healthy lifestyle, more co-morbidities, and lower transportation (i.e., traffic and railway) *L*_night_ levels (data not shown).

The characteristics of the study population included in the final models are summarized in Table 1 and in the Supplemental Material, Table S1. Of all participants, 72.6% did not take any BP-lowering medication (i.e., nonmedicated group). Nonmedicated differed from medicated participants in being younger on average (53 vs. 68 years of age, respectively) but with similar age ranges (35–83 vs. 35–82 years, respectively), having lower BP, higher educational level, fewer co-morbidities, including a greater proportion of women, and smoking and drinking more.

**Table 1:** Main characteristics of the study population (*n* = 3,700) with and without stratification by use of BP-lowering medication.

Variable	Total (*n *= 3,700)	Medicated (*n *= 2,685)	Nonmedicated (*n *= 1,015)	*p*-Value^*a*^
Continuous variables [median (IQR)]
Systolic blood pressure (mmHg)	125 (26.0)	120 (22.0)	139 (27.0)	< 0.001
Diastolic blood pressure (mmHg)	78.0 (13.0)	77.0 (12.0)	81.0 (14.0)	< 0.001
Age (years)	57.0 (20.0)	53.0 (17.0)	68.0 (15.0)	< 0.001
Deprivation index^*b*^	–1.82 (1.28)	–1.82 (1.22)	–1.81 (1.37)	< 0.001
Annual average NO_2_ levels (μg/m^3^)	26.6 (11.7)	26.4 (11.6)	26.8 (11.9)	0.837
Traffic *L*_night_ [dB(A)], 2300–0700 hours	56.6 (7.00)	56.5 (7.00)	56.9 (7.10)	0.022
Railway *L*_night_ [dB(A)], 2300–0700 hours	41.2 (15.2)	41.4 (15.0)	41.0 (15.2)	0.056
Daily mean NO_2_ levels, lag 0 (μg/m^3^)	32.0 (11.9)	32.0 (11.7)	31.7 (12.6)	0.362
Daily mean temperature, lag 0 (ºC)	14.5 (12.4)	14.3 (12.8)	15.0 (11.7)	0.021
Categorical variables [*n* (%)]
Hypertension, yes	1,478 (39.9)	565 (21.0)	913 (90.0)	< 0.001
Sex, male	1,720 (46.5)	1,203 (44.8)	517 (50.9)	0.001
Body mass index
< 20	135 (3.60)	124 (4.60)	11 (1.10)	< 0.001
20–25	1,110 (30.0)	939 (35.0)	171 (16.8)
25.1–30	1,618 (43.7)	1,168 (43.5)	450 (44.3)
> 30	837 (22.6)	454 (16.9)	383 (37.7)
Living alone, yes	413 (11.2)	265 (9.90)	148 (14.6)	< 0.001
Educational level
University or similar	1,050 (28.4)	853 (31.8)	197 (19.4)	< 0.001
Secondary	1,110 (30.0)	878 (32.7)	232 (22.9)
Primary	1,432 (38.7)	902 (33.6)	530 (52.2)
Illiterate	108 (2.90)	52 (1.90)	56 (5.50)
Smoking
Never smokers	1,881 (50.8)	1,329 (49.5)	552 (54.4)	< 0.001
Smokers	811 (21.9)	677 (25.2)	134 (13.2)
Former smokers	1,008 (27.2)	679 (25.3)	329 (32.4)
Diabetes, yes	580 (15.7)	265 (9.90)	315 (31.0)	< 0.001
Daily alcohol intake (g/L)
No alcohol	956 (25.8)	630 (23.5)	326 (32.1)	< 0.001
Little (< 20)	2,237 (60.5)	1,672 (62.3)	565 (55.7)
Moderate (20.1–39.9)	390 (10.5)	292 (10.9)	98 (9.70)
Excessive (≥ 40)	117 (3.20)	91 (3.40)	26 (2.60)
Cardiovascular disease,^*c,d*^ yes	269 (7.30)	83 (3.10)	186 (18.5)	< 0.001
Abbreviations: IQR, interquartile range. ^***a***^Chi-square test and Kruskal–Wallis test for strata of BP-lowering drugs with categorical variables or continuous variables, respectively. ^***b***^High deprivation corresponds to high values. ^***c***^Myocardial infarction, ictus, or any cardiovascular surgical intervention. ^***d***^*n* < 3,700 (< 1% missing observations).				

Home outdoor levels of annual average NO_2_, long-term railway *L*_night_, and traffic intensity at the nearest road were similar in nonmedicated and medicated. The median levels in nonmedicated were 26.4 μg/m^3^, 41.4 dB(A), and 1,400 vehicles/day, respectively. Nonmedicated participants had slightly lower traffic *L*_night_ levels than did medicated participants [56.5 dB(A) vs. 56.9 dB(A), respectively].

The highest correlations of annual mean NO_2_ concentrations were with traffic *L*_night_ (*r* = 0.74) and traffic load within a 500-m buffer (*r* = 0.91) (see Supplemental Material, Table S2).

A 10-μg/m^3^ increase in annual average NO_2_ (from now onward, NO_2_) was associated with a statistically significant increase of 1.15 mmHg (95% CI: 0.34, 1.95) in SBP in nonmedicated in univariate analysis (data not shown) and of 1.34 mmHg (95% CI: 0.14, 2.55) after full adjustment (Table 2). This association was less precise in the group of medicated participants (β = 1.19; 95% CI: –1.37, 3.75). The association between NO_2_ and SBP in the entire population (with and without adjustment for BP-lowering treatment) yielded similar results (β = 1.35; 95% CI: 0.23, 2.47, and β = 1.11; 95% CI: –0.03, 2.24, respectively). Models adding a fixed value of SBP to medicated participants showed smaller estimated effects, steadily shrinking toward the null with increasing fixed values. No association between NO_2_ and SBP was observed with censored regression. The main confounders (i.e., covariates producing the greatest change in point estimates) of the association between NO_2_ and SBP were age, transportation noise (both traffic and railway), and daily temperature. Not adjusting for traffic and railway noise resulted in a smaller coefficient for NO_2_ (β = 0.59; 95% CI: –0.15, 1.34) (Table 2). As additional information, the associations of noise with SBP per 10-dB(A) change in *L*_night_ in the model for nonmedicated participants were β = –0.94 (95% CI: –2.53, 0.64, *p* = 0.244) (traffic noise) and β = –0.21 (95% CI: –0.63, 0.21, *p* = 0.326) (railway noise) (data not shown). The addition of heart rate, CVD, hyperlipidemia, Mediterranean diet, exercise, day of examination, daily temperature, or daily NO_2_ levels at lags other than 0 did not affect results (data not shown). The association between NO_2_ and SBP suggested a positive linear trend > 20 μg/m^3^ (see Supplemental Material, Figure S1). We identified no associations of NO_2_ with DBP (β = 0.15; 95% CI: –0.57, 0.88) or hypertension [odds ratio (OR) = 0.93; 95% CI: 0.79, 1.1] (see Supplemental Material, Tables S3 and S4).

**Table 2:** Estimated effect of a 10-μg/m^3^ increase in annual average home outdoor NO_2_ concentrations [β (95% CI)] on SBP (mmHg).

Models for SBP^*a*^	*n*	Adjusted for noise	Unadjusted for noise
Nonmedicated	2,685	1.34 (0.14, 2.55)	0.59 (–0.15, 1.34)
Medicated	1,015	1.19 (–1.37, 3.75)	0.68 (–1.09, 2.44)
Without adjustment for medication	3,700	1.11 (–0.03, 2.24)	0.56 (–0.17, 1.28)
With adjustment for medication	3,700	1.35 (0.23, 2.47)	0.67 (–0.04, 1.38)
+ 10 mmHg^*b*^	3,700	0.78 (–0.43, 2.00)	0.41 (–0.36, 1.18)
+ 15 mmHg^*b*^	3,700	0.62 (–0.65, 1.89)	0.33 (–0.47, 1.14)
+ 20 mmHg^*b*^	3,700	0.46 (–0.88, 1.80)	0.26 (–0.59, 1.11)
Censored regression	3,700	0.38 (–0.98, 1.73)	0.12 (–0.73, 0.97)
^***a***^All multivariate linear regression models were adjusted for age, age squared, sex, living alone, education, diabetes, BMI, nighttime railway noise, nighttime traffic noise, smoking, alcohol consumption, deprivation, daily NO_2_, and temperature (lag 0). Models additionally were adjusted for noise (nighttime railway and traffic noise) and BP-lowering medication if specified in table. ^***b***^Addition to SBP for participants with BP-lowering medications.			

**Figure 1:**
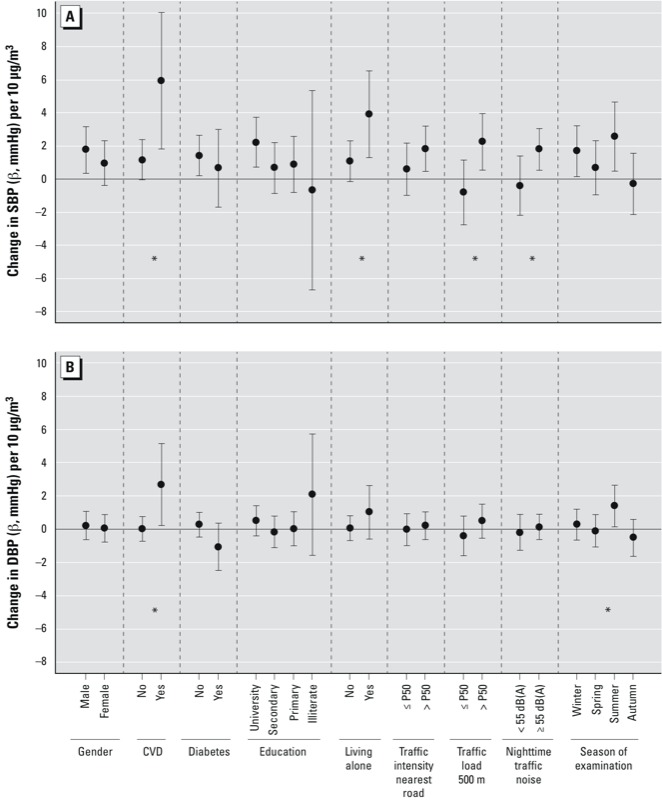
Adjusted β coefficients and 95% CIs for the association of (*A*) SBP and (*B*) DBP (mmHg) blood pressure with a 10-μg/m^3^ increase in annual average home outdoor NO_2_ concentrations by subgroups of the population (*n* = 2,685 nonmedicated participants). Each multivariate linear regression model was adjusted for the corresponding interaction term, one at a time, and age, age squared, sex, living alone, education, diabetes, BMI, nighttime railway noise, nighttime traffic noise, smoking, alcohol consumption, deprivation, daily NO_2_, and temperature (lag 0). P50, 50th percentile. *p for interaction < 0.05.

Figure 1 shows the interaction analyses for the association between NO_2_ and SBP and DBP, respectively, in nonmedicated participants. NO_2_ was more strongly associated with SBP among individuals with CVD (β = 5.96; 95% CI: 1.85, 10.08) than among individuals without (β = 1.17; 95% CI: –0.04, 2.38), *p* for interaction = 0.02. With DBP there was evidence of an association only among individuals with CVD (β = 2.71; 95% CI: 0.23, 5.18, *p* for interaction = 0.03). A stronger association between NO_2_ and SBP was also found in those living alone (β = 3.93; 95% CI: 1.32, 6.55) than for those living with more people (β = 1.10; 95% CI: 0.23, 5.18), *p* for interaction = 0.03. NO_2_ was associated with SBP in participants exposed to higher traffic levels, particularly to traffic loads above the median within 500-m buffer (β = 2.28; 95% CI: 0.58, 3.97) and not in those exposed to lower levels (β = –0.79; 95% CI: –2.73, 1.15), *p* for interaction = 0.007. This was also the case for individuals exposed to traffic *L*_night_ ≥ 55 dB(A) (β = 1.82; 95% CI: 0.56, 3.07) compared with those exposed to lower noise levels (β = –0.39; 95% CI: –2.17, 1.39), *p* for interaction = 0.03. Associations of NO_2_ with SBP and DBP were stronger among participants whose BP was measured in summer versus other seasons, and this seasonal interaction was statistically significant for DBP only (*p* for interaction = 0.04). Sex, educational level, and diabetes (Figure 1), and age, smoking, and Mediterranean diet (data not shown) did not modify the main associations. Interaction analyses showed the same patterns when a fixed value of mmHg was added to the measured BP levels of medicated subjects, or when censored regression was used to control for medication use (data not shown).Finally, there was no statistical evidence of differences in the associations for the nonmedicated and medicated groups (Table 2; see also Supplemental Material, Table S3) neither with SBP nor with DBP (*p* for interaction = 0.472 and 0.318, respectively; interactions not shown).

In a subsample with residential history, restricting the sample to nonmovers or using 10-year average NO_2_ levels yielded similar or slightly smaller increases in BP in nonmedicated participants compared with findings for the annual average NO_2_ levels at the current address (see Supplemental Material, Table S5).

## Discussion

This is one of few studies to analyze the association between near-road traffic-related air pollution and both blood pressure (BP) and hypertension, and to control for detailed transportation noise information. Moreover, in this study we evaluated in detail the influence on findings of using different methodologies to control for BP-lowering medication.

This cross-sectional population-based study showed a statistically significant association between long-term exposure to home outdoor NO_2_, a widely used marker of near-road traffic-related air pollution, and SBP among participants not taking BP-lowering medication (nonmedicated). This association was similar in the entire population whether adjusting or not for medication, although the latter association was slightly weaker. When using methods proposed to better account for medication in the entire sample (Tobin et al. 2005)—namely, methods *e* fixed addition and *f* censored regression—the relationship was weaker or diluted. Associations were adjusted for exposure to transportation noise, short-term air pollution levels, and temperature, and were robust to the inclusion of several covariates (data not shown). No significant associations were generally found for hypertension or DBP, although we observed associations with DBP in some subgroups of the population.

*Blood pressure*. The main results for SBP are consistent with most of the few studies available. Fuks et al. (2011) and Schwartz et al. (2012) reported estimated effects with urban background particulate matter with aerodynamic diameter ≤ 2.5 μg/m^3^ (PM_2.5_) and black carbon, respectively, but they did not analyze NO_2_. Regarding NO_2_, one study reported inverse associations between nitrogen oxides (NO_x_, NO, and NO_2_) and SBP (Sørensen et al. 2012); a study in China reported no association (Dong et al. 2013); and a survey of elderly in Taiwan (Chuang et al. 2011) reported stronger estimated effects than ours, namely a 11.22-mmHg increase in SBP (95% CI: 8.56, 13.89) per 10 μg/m^3^. Differences among studies may reflect different population characteristics (e.g., age, ethnicity, lifestyle) or residual confounding. Moreover, to the extent that NO_2_ may serve as a marker, differences in the air pollution mixtures between the study areas may also explain the discrepancies.

As reported in two previous epidemiological studies, there was no association between NO_2_ and DBP (Auchincloss et al. 2008; Sørensen et al. 2012). Animal and human experimental studies suggest that BP responses to long-term exposure to air pollution may be mediated by sustained systemic inflammation and/or oxidative stress, impairing endothelial function and increasing BP (Brook et al. 2009; Mills et al. 2008). As discussed by Auchincloss et al. (2008), endothelial dysfunction and subsequent stiffening of the aorta would result in increased SBP but lower DBP (i.e., increased pulse pressure), and subsequent isolated systolic hypertension after 60 years of age (Franklin 2006).

*Effect of BP-lowering treatment*. One of the difficulties in studying BP is that antihypertensive medication is a potential mediator between air pollution and measured BP levels, and not a confounder of the studied association. Consequently, the common procedures of adjustment for medication may introduce bias. Previous studies analyzed nonmedicated participants (Sørensen et al. 2012), did not adjust for medication (Chuang et al. 2011), adjusted for medication (Schwartz et al. 2012), or compared models with and without adjustment (Fuks et al. 2011). To overcome this problem, some methodologies have been proposed (Tobin et al. 2005). However, as discussed below, the latter also rely on nonmeasurable assumptions that may be violated in some populations.

In the nonmedicated group, we observed a statistically significant positive association between NO_2_ and SBP, whereas no association was observed between NO_2_ and BP in medicated participants. These results may agree with the hypothesis that effects of air pollution on BP can be masked by medication, and that the association might be more clearly observed in the nonmedicated group. Conditioning (i.e., stratifying) on medication can lead to collider stratification bias if air pollution is associated with medication, and a third factor is associated with both medication and measured BP. NO_2_ levels were similar in medicated and nonmedicated in the entire population (Table 1) and among hypertensive participants, a fact that may minimize the potential for this bias. The main predictors of being nonmedicated but with hypertension were being younger and healthier, but NO_2_ was not associated. However, younger age, which was associated with lower BP levels, also was associated with lower NO_2_ levels (Spearman rank *r* = 0.11, *p* < 0.001). Although our multivariate models took all these factors into account, residual bias mediated through age or due to unmeasured characteristics cannot be excluded.

As suggested by Fuks et al. (2011), if air pollution leads to high BP, medication intake, and subsequent decreases in measured BP levels, and if this is the only mechanism in place, then we should observe an inverse association between air pollution and measured BP levels. However, given the wide range of the population studied, different treatments potentially used, and likely diverse compliance with treatment, heterogeneity in the treatment effects is expected. This may produce not an inverse relationship but a positive but underestimated one in medicated participants and in the entire population without adjustment for treatment. Under the assumption of heterogeneity, medication would also be a weaker mediator of the association between NO_2_ and BP, thus resulting in less bias when adjusting for medication.

Using a fixed addition of 10 mmHg in SBP for those treated led to a positive though weaker and not statistically significant association, whereas larger additions showed a steady shrinkage of results toward the null. As suggested by McClelland et al. (2008), in many scenarios the absolute treatment effect will depend on the underlying BP levels and the target therapeutic values to achieve for BP; thus, the simple addition of a fixed value would be inadequate. The difference in BP levels before treatment and during treatment may depend on several characteristics (e.g., SES) or unmeasured factors that can in turn be associated with exposure. This can also bias the results for the fixed addition method. Regarding censored regression, one of the assumptions of this model is that the distribution of underlying BP in treated subjects is the same as the distribution of measured BP in untreated subjects, which might be often wrong (Tobin et al. 2005) and not necessarily true in our sample. This methodology can also underestimate or overestimate the truth (McClelland et al. 2008).

In summary, no perfect methodology exists to account for BP-lowering medication, because all approaches rely on assumptions regarding the effect of medication that may vary across scenarios and introduce bias. The heterogeneity in the treatment effect may reduce bias that might be introduced by commonly used procedures to account for the effect of medication use. In light of the nontrivial differences in estimated effects across the various methods, more studies are needed to clarify the complex conceptual challenge we raise in this study.

*Hypertension*. Prevalence of hypertension was not associated with annual average NO_2_ concentrations in our study. The few previous studies found inconsistent results for prevalence of hypertension with different pollutants (Dong et al. 2013; Fuks et al. 2011; Johnson and Parker 2009; Sørensen et al. 2012). Regarding incidence of hypertension, no association was found in Denmark (Sørensen et al. 2012), whereas another study reported an increased risk of hypertension that was statistically significant with long-term NO_2_ but not with PM_2.5_ (Coogan et al. 2012).

We hypothesized the differences to be attributable to misclassification of hypertensive cases, as proposed before (Fuks et al. 2011). However, the different definitions of hypertension yielded all null results (see Supplemental Material, Table S4). Alternatively, the null associations may relate to a loss in statistical power, but also of information, when using the binary variable of hypertension. In fact, we observed a nonsignificant increased OR when assessing prehypertension and hypertension together. Prehypertensives are nonmedicated individuals with suboptimal BP levels not reaching the cut-off for hypertension. The assessment of this group, which has increased CV risk (Chobanian et al. 2003), might also be informative for the effects of air pollution. Results considering this nonmedicated group agree with results on BP and may explain findings among nonmedicated. However, by reclassifying prehypertensive participants, we are partially reclassifying by medication use. Thus, as explained for the BP analyses among nonmedicated, residual stratification collider bias cannot be excluded.

*Transportation noise*. Road traffic and railway noise have been associated with high BP (Dratva et al. 2012; van Kempen and Babisch 2012; Sørensen et al. 2011). Therefore, we considered transportation noise as a potential confounder. Although traffic noise and NO_2_ were highly correlated (*r* = 0.74), the negative confounding of transportation noise on the studied association with SBP was not explained by collinearity among these factors [mean variance inflation factor (VIF) for the model = 1.45, VIF for NO_2_ = 3.2, individual VIF for road *L*_night_ = 2.25]. We hypothesized that the negative confounding might be explained partly by the use of protections against noise among participants more exposed to noise (e.g., closing windows at night, sleeping in a room facing the backyard). Thus, the estimates of outdoor noise may not be sufficient to investigate the independent effects of this stressor on BP in our setting. In Girona, people live close to traffic, and traffic noise is prevalent. Moreover, complaints about neighborhood noise are generally high in Spain. Thus, participants may more likely use remedies against noise in our study area than in settings with less local traffic or different urban structures. Protections are expected to affect noise exposure to a greater extent than air pollution, because noise propagation depends on physical barriers, whereas air pollution distributes more evenly and its infiltration rates are high (Chen and Zhao 2011). Moreover, because noise is subject to annoyance, the protective behavior will more likely correlate with noise than air pollution exposure. We consider better noise exposure assessment a crucial next step to elucidate the role of noise. Thus, we are currently developing methods to estimate individual exposure to traffic-related noise levels indoors at homes.

*Effect modification*. We observed a stronger positive association between NO_2_ and BP in participants with CVD, similar to Sørensen et al. (2012), who reported this interaction between long-term residential NO_x_ and DBP. However, given the small numbers in both cases, further studies are needed that investigate this interaction. We also found a stronger association for participants living alone. Living alone was strongly correlated with being older and exposed to slightly higher NO_2_ levels in our population, and Morgado et al. (2010) identified it as a predictor of uncontrolled hypertension among hypertensives (Morgado et al. 2010).

The association between NO_2_ and SBP was stronger in those more exposed to traffic and road traffic noise [≥ 55dB(A)]. This may be attributable to nonlinearity in the estimated effects of NO_2_ on BP. Indeed, we observed a null to negative association at very low NO_2_ levels (compared with levels in larger Spanish and European cities) and a clear positive trend > 20 μg/m^3^ (see Supplemental Material, Figure S1). Our results did not change by the use of a quadratic term (data not shown), because the nonlinearity was observed at very low levels, and our sample was distributed across a wider range of NO_2_ concentrations. However, the observation of nonlinearity may suggest that, at higher traffic levels, NO_2_ may be more representative of near-road traffic-related pollutants, which are particularly associated with CVD (Auchincloss et al. 2008; Brook et al. 2009; Hoffmann et al. 2007; Künzli et al. 2010). In our study areas, low levels of NO_2_ reflect sites with little traffic, so exposure is mostly related to urban background pollution; however, in other urban areas, NO_2_ is clearly a marker for near-road traffic-related pollutants. Similar reasons may explain the negative associations observed between SBP and NO_x_ in Denmark (Sørensen et al. 2012), where many individuals were exposed to particularly low NO_2_ levels (median; 5th–95th percentile: 16.3; 12.0–32.6, at baseline) compared with those in our study area (26.6; 12.6–39.6). In addition, the association between NO_2_ and SBP tended to the null when adjusting for traffic intensity or traffic load within 500 m (results not shown), which also suggests that we are observing associations for traffic-related air pollution, rather than for background pollution.

*Strengths and limitations*. The main limitation of this study was its cross-sectional design. Our results should be confirmed in longitudinal analysis with repeated measures of BP and incidence of hypertension.

As in many epidemiological studies, BP was measured with standard protocols, consisting in repeated measures taken during one single examination, not following clinical procedures to diagnose hypertension. Although results were robust to the different definitions of hypertension, and we selected the last BP measurement available to minimize the “white-coat effect,” a residual misclassification of the outcome cannot be excluded. Because random error in BP measures can be expected, the misclassification could likely be nondifferential, biasing results toward the null.

Regarding exposure misclassification, we assessed individual residential outdoor exposure, not personal exposure. Although people spend a relevant part of their time at home, as observed in different European areas and climates (Schweizer et al. 2007), we cannot conclude whether time–activity patterns in our population would affect our findings. Although our LUR model was developed after the study examinations were completed, LUR models are good predictors of spatial gradients over time (Eeftens et al. 2011). Moreover, no major changes in traffic or monitored background NO_2_ levels occurred from 2003 to 2009 (data not shown). Finally, we explored whether past exposure influenced the studied associations with BP levels in a subsample with residential history. Although the subsample was not fully representative of the study population (slightly younger group with lower BP levels), results in this subsample were not affected by past residential mobility or by using a longer time window of exposure.

Another limitation was the lack of information on lead exposure from leaded gasoline used before 2000 in Spain. Long-term cumulative exposure, reflected by bone lead levels, could remain high in this adult population and potentially interact with or confound the association between NO_2_ and high BP (Schwartz et al. 2011).

As an important strength, we evaluated a population-based cohort and a wider age range compared with previous study populations, some of which had only elderly participants (Chuang et al. 2011; Schwartz et al. 2012). Thus, our results in the entire population can be generalized. Additional strengths include the comprehensive analysis of BP-lowering medication, the use of a LUR model that captured the intraurban variability in NO_2_ levels and that allowed individual exposure assessment, and the control for many covariates, including detailed transportation noise data, which has rarely been taken into account in previous literature (Fuks et al. 2011; Sørensen et al. 2012).

## Conclusions

We observed a positive association between long-term exposure to NO_2_ and SBP in a population-based cohort in Girona, after adjustment for transportation noise. The association was sensitive to the selected methodology to control for BP-lowering medication, and was stronger among participants with CVD, those living alone, and those exposed to more traffic and road traffic noise. These results suggest specific effects of near-road traffic-related pollutants on BP. High BP might be a pathway through which air pollution causes CVD. Although the estimated effect size was small, these findings are relevant for public health, given the ubiquity of air pollution, which affects millions of people. Indeed, a “small” reduction of 2 mmHg in the population mean SBP has been estimated to result in a 25% reduction in stroke events (Girerd and Giral 2004).

## Supplemental Material

(172 KB) PDF
