# Supplementary material for: Association of Long-Term Exposure to Traffic-Related Air Pollution with Blood Pressure and Hypertension in an Adult Population–Based Cohort in Spain (the REGICOR Study)
Source: Environ Health Perspect. 2014 Feb 14;122(4):404–11. doi: 10.1289/ehp.1306497 (PMC3984222; doi:10.1289/ehp.1306497)
Supplement: (172 KB) PDF [file ehp.1306497.s001.pdf]

Environmental Health Perspectives doi:10.1289/ehp.1306497

Supplemental Material

Association of Long-Term Exposure to Traffic-Related Air Pollution with Blood Pressure and Hypertension in an Adult Population–Based Cohort in Spain (the REGICOR Study)

Maria Foraster, Xavier Basagaña, Inmaculada Aguilera, Marcela Rivera, David Agis, Laura Bouso, Alexandre Deltell, Jaume Marrugat, Rafel Ramos, Jordi Sunyer,[1,2,3] Joan Vila,[2,7] Roberto Elosua, and Nino Künzli

Table of Contents	Page
**Table S1**	2
**Table S2**	3
**Table S3**	4
**Table S4**	5
**Table S5**	6
**Figure S1**	7

Table S1. Extension of characteristics of the study population (N = 3700) reported in Table 1, with and without stratification by use of blood pressure (BP)-lowering medication.

Characteristic	Total (N = 3700)	No medication (N = 2685)	Used medication (N = 1015)	p-value[a]
**Continuous variables [median (IQR)]**				
Weekly physical activity (MET)	1519 (1886)	1491 (1828)	1575 (1952)	0.218
Mediterranean diet adherence score [b]	20.0 (4.00)	20.0 (4.00)	20.0 (4.00)	0.136
Traffic intensity in nearest road (veh/day)	1459 (5674)	1400 (5662)	1459 (5466)	0.192
Traffic load within 500 m buffer/10000 (veh·m/day)	8043 (6539)	8014 (6461)	8125 (6653)	0.807
**Categorical variables [N (%)]**				
Occupational status[c], working	2003 (54.4)	1721 (64.4)	282 (27.9)	< 0.001
Homemaker and economically inactive	479 (13.0)	319 (11.9)	160 (15.8)	
Retired	1117 (30.3)	560 (21.0)	557 (55.1)	
Unemployed	84 (2.30)	73 (2.70)	11 (1.10)	
Heart rate[c], < 60	626 (16.9)	432 (16.1)	194 (19.2)	< 0.001
60-80	2584 (69.9)	1922 (71.7)	662 (65.4)	
80.1-100	459 (12.4)	317 (11.8)	142 (14.0)	
> 100	26 (0.70)	11 (0.40)	15 (1.50)	
Direct relative died for cardiac reasons[c], yes	366 (10.0)	251 (9.40)	115 (11.5)	0.064
Hyperlipidemia[c,d], yes	1067 (28.9)	661 (24.6)	406 (40.0)	< 0.001
Season, winter	1065 (28.8)	810 (30.2)	255 (25.1)	0.025
Spring	1242 (33.6)	886 (33.0)	356 (35.1)	
Summer	664 (17.9)	475 (17.7)	189 (18.6)	
Autumn	729 (19.7)	514 (19.1)	215 (21.2)	

[a]χ^2 test and Kruskal-Wallis test for strata of BP-lowering drugs with categorical variables or continuous variables, respectively. [b]10 (lowest) and 30 (highest) adherence to diet. [c]N below 3700 (< 1% missing observations). [d]Hyperlipidemia defined as having total cholesterol > 250 mg/dl or taking statins or any treatment to decrease cholesterol levels.

Table S2. Spearman's correlation between the long-term and short-term environmental factors (N = 3700).

**Variables**	**Annual NO_2**	**Traffic L_{night}**	**Railway L_{night}**	**Daily NO_2, lag 0**	**Daily temperature, lag 0**	**Traffic intensity**
Annual NO_2 ($\mu g/m^3$)	1.00					
Traffic L_{night} (dB(A))	0.74*	1.00				
Railway L_{night} (dB(A))	0.66*	0.48*	1.00			
Daily NO_2, lag 0	0.02	-0.02	0.03*	1.00		
Daily temperature, lag 0	0.02	-0.04*	-0.10*	-0.20*	1.00	
Traffic intensity nearest road (veh/day)	0.62*	0.76*	0.37*	-0.03	-0.04*	1.00
Traffic load within 500 m buffer (veh·m/day)	0.91*	0.59*	0.71*	0.02	0.02	0.49*

NO_2: nitrogen dioxide; L_{night}: long-term average nighttime noise levels.

*p-value < 0.05.

Table S3. Estimated effect of a 10-µg/m^3 increase in annual average home outdoor NO_2 concentrations and 95% confidence intervals on diastolic blood pressure (DBP).

Models for DBP	N	beta (95% CI)[a]	beta (95% CI)[b]
Non-medicated	2685	0.15 (-0.57, 0.88)	0.12 (-0.33, 0.57)
Medicated	1015	0.66 (-0.67, 1.99)	0.53 (-0.39, 1.44)
Without adjustment for medication	3700	0.22 (-0.43, 0.87)	0.23 (-0.18, 0.64)
With adjustment for medication	3700	0.33 (-0.32, 0.97)	0.28 (-0.13, 0.69)
+5 mmHg[c]	3700	0.06 (-0.62, 0.74)	0.15 (-0.28, 0.59)
+10 mmHg[c]	3700	-0.10 (-0.83, 0.64)	0.08 (-0.39, 0.55)
+15 mmHg[c]	3700	-0.26 (-1.07, 0.55)	0.004 (-0.51, 0.52)
Censored regression	3700	-0.29 (-1.07, 0.48)	-0.08 (-0.56, 0.41)

[a]Multivariate linear regression models, adjusted for: age, age squared, sex, living alone, education, diabetes, BMI, nighttime railway noise, nighttime traffic noise, smoking, alcohol consumption, deprivation, daily NO_2 and temperature (lag 0). [b]Multivariate linear regression models adjusted for covariates in [a] except for nighttime railway and traffic noise. [a] and [b] adjusted for BP-lowering medication if specified in table. [c]Addition to DBP for participants with BP-lowering medications.

Table S4. Estimated effect of a 10-μg/m^3 increase in annual average home outdoor NO_2 concentrations and 95% confidence intervals on the prevalence of hypertension and prehypertension.

Models for hypertension (HT) [percentage of cases]	N	OR (95% CI) [a]	OR (95% CI) [b]
HT main outcome [40.0%] [c]	3700	0.93 (0.79, 1.10)	1.00 (0.90, 1.11)
HT alternative outcome [42%] [d]	3700	0.98 (0.83, 1.15)	1.00 (0.90, 1.11)
HT extremes [38%] [e]	3101	0.91 (0.75, 1.09)	0.95 (0.84, 1.07)
HT or prehypertension [67.8%] [f]	3700	1.10 (0.93, 1.32)	1.04 (0.94, 1.16)

[a] Multivariate logistic regression models, adjusted for: age centered, sex, living alone, education, diabetes, BMI, deprivation, daily NO_2 and temperature (lag 0), nighttime traffic noise, nighttime railway noise. [b] Multivariate logistic regression models adjusted for covariates in [a] except for nighttime railway and traffic noise. [c] Hypertension defined as having SBP or DBP ≥ 140/90 mmHg, respectively, or as a positive response to the question "Do you take or have you taken any doctor prescribed medication to reduce blood pressure in the last two weeks?" [d] Hypertension defined as having SBP or DBP ≥ 140/90 mmHg, respectively, or reporting antihypertensive-like treatment in the medication list provided by participants and coded by a physician into "antihypertensive" or "beta-blocker" (i.e., diuretics, ACE inhibitors, alpha or beta-blockers, angiotensin receptor II blockers, and calcium channel blockers). [e] Same definition as [c] excluding participants with borderline BP levels and not reporting use of antihypertensive treatment, i.e. with SBP and DBP ≥ 135/85 mmHg and < 150/95 mmHg. [f] Hypertension or prehypertension defined as having SBP or DBP ≥ 120/80 mmHg, respectively, or as a positive response to the question "Do you take or have you taken any doctor prescribed medication to reduce blood pressure in the last two weeks?"

Table S5. Estimated effect of a 10-µg/m^3 increase in annual average home outdoor NO_2 concentrations and 95% confidence intervals at the current address, for non-movers and for 10-year average home outdoor NO_2 concentrations on systolic (SBP) and diastolic (DBP) blood pressure. Non-medicated population with residential history (N = 1843).

**Model**	**N**	**SBP: beta (95%CI)[a]**	**DBP: beta (95%CI)[a]**
Annual NO_2 at current address	1843	2.11 (0.66, 3.57)	0.72 (-0.16, 1.61)
10-y average NO_2	1843	1.51 (0.14, 2.88)	0.40 (-0.43, 1.23)
Non-movers 2 years	1754	1.98 (0.48, 3.47)	0.73 (-0.18, 1.63)
Non-movers 5 years	1575	2.02 (0.43, 3.61)	0.89 (-0.06, 1.83)
Non-movers 10 years	1219	1.93 (0.02, 3.84)	0.90 (-0.21, 2.00)

[a]Multivariate linear regression models adjusted for: age, age squared, sex, living alone, education, diabetes, BMI, nighttime railway noise, nighttime traffic noise, smoking, alcohol consumption, deprivation, daily NO_2 and temperature (lag 0).

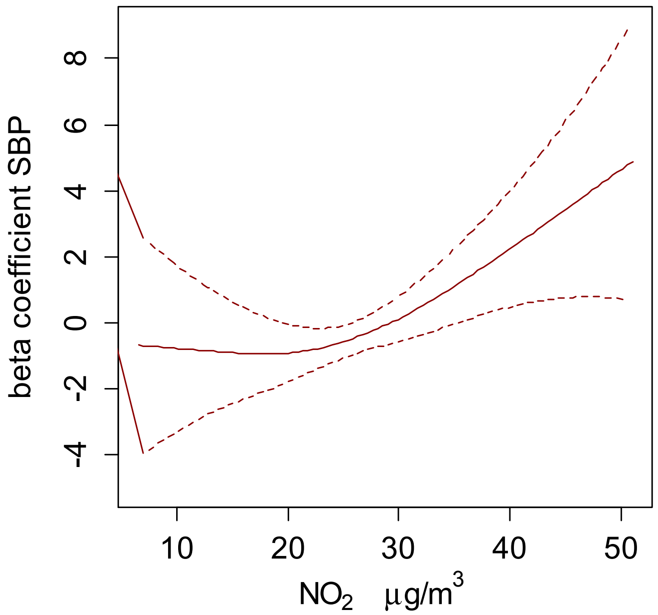

Figure S1. Smooth spline and 95% confidence intervals for the association between annual average home outdoor NO_2 levels (µg/m^3) and systolic blood pressure (SBP, mmHg), (N = 2685, non-medicated participants). Generalized additive model adjusted for: age, age squared, sex, living alone, education, diabetes, BMI, nighttime railway noise, nighttime traffic noise, smoking, alcohol consumption, deprivation, daily NO_2 and temperature (lag 0).
